# Impact of light activation on tooth whitening using three different light sources

**DOI:** 10.6026/9732063002001334

**Published:** 2024-10-31

**Authors:** Raktim De, Rehan Ahmad Khan, Aditya Singh, Keshav Manglam, Syed Zaki Anwar, Samra Shafique, Shazia Siddiqui

**Affiliations:** 1 Department of Conservative Dentistry & Endodontics, Mithila Minority Dental College & Hospital, Darbhanga, Bihar, India; 2 Department of Conservative Dentistry & Endodontics, Sardar Patel Postgraduate Institute of Dental and Medical Science, India; 3 Endodontist, Autonomous State Medical College, Bahraich, India; 4 Department of Conservative Dentistry & Endodontics, Career Postgraduate Institute of Dental Science and Hospital, Lucknow, India

**Keywords:** Blue-phase N, Dental Bleaching, mcinnes solution, LED light, VITA shade guide, plasma arc-based light

## Abstract

Tooth discolorations, whether caused by external factors, internal factors, or the individual's own acceptance of the discoloration, are often intricate in nature. The white colour of adult teeth fades as a result of alterations in the enamel and dentin. Dental aesthetics heavily relies on tooth shade. Vital teeth whitening is a much sought-after cosmetic dental procedure aimed at enhancing one's smile. Hence, our objective was to assess the effectiveness of three distinct light sources employed in dental whitening treatments. A total of 20 patients were chosen who did not have any tooth decay or sensitivity in the dentin for our trial. The VITA shade guide was used for pre-operative shade choosing. The group has been broken into four groups, namely Group A, Group B, Group C and Group D. The McInnes solution was used to treat the tooth surface in Group A. No light activation was done. In Group B, after the application of McInnes solution, it was activated with an LED light. Following the application of McInnes solution, Group C was subsequently activated using Blue-phase N. In Group D, the McInnes solution was applied and subsequently activated using plasma arc-based light, specifically red-blue light. All the assessed groups demonstrated some level of whitening effect, but the group that used plasma arc-based light showed a more pronounced change in shade rank compared to the other groups. The mean difference in shade rank between the pre-operative and 1-month post-operative stages was 2.20 for Group A, 3.40 for Group B, 5.80 for Group C, and 9.60 for Group D (p=0.026). Similarly, the mean difference in shade rank between the pre-operative and 6-month post-operative stages was 0.00 for Group A, 2.60 for Group B, 3.60 for Group C, and 9.60 for Group D (p=0.011). The Kruskal-Wallis test revealed significant differences among the groups at both the 1-month and 6-month post-operative stages. It was discovered that the type of illumination used in dental bleaching greatly affects the outcome, with some lights producing better long-term results than others. The data also show that specific light sources may boost the efficacy of tooth bleaching operations, warranting a personalised strategy depending on the light's performance over time.

## Background:

The pursuit for an aesthetically pleasing smile has long been a vital element of dental medicine, with tooth whitening being one of the most sought-after cosmetic dentistry operations. Historically, the quest of dental aesthetics may be dated back to 1877 when the concept of tooth bleaching was first reported [1]. The evolution of bleaching procedures has witnessed important innovations, from the use of hydrochloric acid by Dr. Walter Kane in 1916 to the introduction of hydrogen peroxide as a safer and more effective bleaching agent by Ames in 1937 [2]. Today, the enhancement of dental aesthetics through bleaching is not only a reflection of personal health and cleanliness but also a technique strongly ingrained in the cultural framework of beauty. Dental whitening techniques have varied to include at-home approaches, over-the-counter items and professionally performed in-office treatments. Among these, in-office bleaching is particularly beneficial for its speedy results and effectiveness in cases of severe discolouration and when patient compliance is doubtful [3]. This approach relies on the controlled application of high-concentration peroxide agents, frequently activated by various light sources to speed the bleaching process. The light sources widely utilised include plasma-arc, light-emitting diode (LED), metal halide, argon laser, and xenon halogen, each considered to promote the decomposition of hydrogen peroxide, hence speeding up the whitening process [4]. The underlying mechanism of action for teeth whitening is the passage of hydrogen peroxide into the enamel and dentin, where it reacts with organic molecules. The resultant chemical reactions release radicals that disturb the conjugation of electron systems within the organic molecules of tooth enamel. This disruption changes the light absorption capabilities of the enamel, effectively resulting to a whitened look [5]. The ultimate goal in modern dentistry treatment is to obtain optimal aesthetic outcomes while conserving the natural tooth structure as much as possible, frequently making bleaching a preferable alternative over more invasive procedures like veneers or crowns [6, 7-8]. Given the range of light sources used in dental bleaching and the lack of consensus on their comparative efficiency, this randomized clinical trial was designed to examine three different light sources within the context of in-office dental bleaching. By assessing the efficacy of these technologies, the study seeks to contribute to the optimization of bleaching techniques, assuring both the aesthetic satisfaction of the patients and the preservation of dental health.

## Materials and Methods:

## Study design:

This randomized control trial was conducted at the Career Post Graduate Institute of Dental Sciences and Hospital in Lucknow, Uttar Pradesh, India, which served as the primary site for participant recruitment, treatment, and follow-up. The study was designed in compliance with the institutional ethical committee's codes of research ethics and obtained the IEC certificate with the number CPGIDSH/22/274.

## Selection criteria:

Participants aged 18-40 years, both males and females, were selected for the study. The study focused on maxillary anterior teeth with intrinsic stains such as fluorosis and amelogenesis imperfecta. However, patients with dentinal hypersensitivity, pregnancy, lactation, and periapical pathology were excluded from the study.

## Sample size and procedure:

Twenty patients without caries and dentinal hypersensitivity were selected for the study. Pre-operative shade selection was performed using the VITA shade guide. Rubber dam isolation was carried out, and a gingival tissue barrier was applied over the dam. McInnes solution (Prevest Denpro), which consists of 5 parts 30% H2O2, 5 parts 36% HCl and 1 part anaesthetic ether/alcohol, was mixed in a dapen dish and applied to the labial surface of the tooth.

## Treatment groups:

The patients were divided into four groups, with five patients in each group. In Group A, McInnes solution was applied to the tooth surface without light activation. In Group B, McInnes solution was applied to the tooth surface and activated with LED light. In Group C, McInnes solution was applied to the tooth surface and activated with Blue-Phase N light. In Group D, McInnes solution was applied to the tooth surface and activated using plasma arc-based light (Red-Blue light). In all groups, the solution was applied three times with 20-minute intervals, and post-operative shade selection was taken using the VITA shade guide after the final application. In Group D, the plasma arc-based light was activated in three cycles: the first cycle with Blue light for 10 minutes, the second cycle with Red light for 10 minutes, and the third cycle with Red-Blue light for 10 minutes. In cases of severe fluorosis, microabrasion was performed with pumice mixed with HCl using a slow-speed handpiece and rubber cup attachment after one round of the cycle.

## Statistical analysis:

We analysed bleaching outcomes using the VITA shade guide, summarizing the data as Mean ± SD and proportions. We employed Statistical Package for Social Sciences, version 18 (SPSS Inc., Chicago, IL) and MS Excel for our statistical analyses. The non-parametric Kruskal Wallis test was used for comparing results across different groups, while the Wilcoxon test was utilized for analysing changes within each group over time. Additionally, we used the chi-square test to examine the distribution of categorical data. P-value > 0.05 was taken to be significant level.

## Results:

The Distribution of Study Subjects by Pre-Procedure shade Rank is shown in Table 1. The subjects had maximum rank of 15 and minimum 5. The Distribution of Study Subjects by 1 month Post Procedure Rank is shown in Table 2. The subjects had maximum rank of 9 and minimum 1 at this stage. The Distribution of Study Subjects by 6 month Post Procedure Rank is shown in Table 3. The subjects had maximum rank of 9 and minimum 1 at this stage. The Intergroup Comparison of Pre-Procedure Shade Rank showed the mean rank of group A was 8.80±2.49, in group B it was 8.60±4.10, for group C it was 10.80±2.68 and for group D it was 14.20±2.95. No significant difference was found in mean shade rank among the groups (p=0.057). The Intergroup Comparison of 1 month Post Procedure Shade Rank showed the mean rank of group A was 6.60±2.19, in group B it was 5.20±2.49, for group C it was 5.00±2.83 and for group D it was 4.60±0.89. No significant difference was found in mean shade rank among the groups (p=0.477) (Table 4, Table 5).

Figure 1 - see PDF shows the Intergroup Comparison of 6-month Post Procedure Shade Rank showed the mean rank of group A was 8.80±2.49, in group B it was 6.00±3.00, for group C it was 7.20±4.27 and for group D it was 4.60±0.89. No significant difference was found in mean shade rank among the groups (p=0.155).

Figure 2 - see PDF shows the Intergroup Comparison of Differences of Pre-Procedure. To 1 month Post Procedure Shade Ranks showed the mean rank difference of group A was 2.20±2.05, in group B it was 3.40±4.10, for group C it was 5.80±3.03 and for group D it was 9.60±3.36. The significant difference was found in mean shade rank difference among the groups (p=0.026). Further greater difference showed superior quality of the group. Hence the groups can be arranged according to their quality as

Group D > Group C > Group B > Group A

Figure 3 - see PDF shows the Intergroup Comparison of Differences of Pre Proc. To 6 month Post Procedure Shade Ranks showed the mean rank difference of group A was 0.00±0.00, in group B it was 2.60±4.34, for group C it was 3.60±4.98 and for group D it was 9.60±3.36. The significant difference was found in mean shade rank difference among the groups (p=0.011). Further greater difference showed superior quality of the group. Hence the groups can be arranged according to their quality as

Group D > Group C > Group B > Group A

## Discussion:

Potential side effects, like superficial tooth enamel decalcification and increased dental sensitivity, have been closely examined in relation to the use of chemicals in dental bleaching. This discomfort results from the breakdown of the pigments that discolour teeth by the nascent oxygen produced. It has been suggested that within a few days, enamel integrity can be restored through the natural remineralization process, which is aided by the calcium in saliva or the administration of fluoride gel after bleaching [5]. Violet light bleaching offers a novel method of teeth whitening because it does not use peroxide gels. Because of its intermittent application technique, this technology successfully dissolves pigments without generating demineralization or heat-induced sensitivity by relying on the intrinsic qualities of light. In terms of bleaching efficacy, an immediate evaluation post-treatment indicated that the use of a Power bleaching light (Group D) achieved a more pronounced whitening effect compared to other groups. Conversely, the McInnes solution (Group A), used without light activation, showed minimal color changes with observable relapse over time. Group C (Blue-phase N) maintained a lighter shade for up to a month post-treatment, but a regression to a darker shade was noted after six months. Statistical analysis revealed no significant differences in color stability between groups using and not using LED light, with similar relapse patterns noted across the groups after one and six months.

Marson *et al.* [9] found no significant difference in color stability between groups after 6 months, suggesting that hydrogen peroxide concentration and light activation may not impact long-term color stability. In contrast, Matis *et al.* [10] reported a reversal of color regardless of concentration and light activation. Our study's results parallel those of Bhutani *et al.* [11] who observed a slight improvement in the group with light activation, hinting at a beneficial effect of light activation on tooth whitening. However, Sabnis *et al.* [12] found no difference between light-activated and chemically activated bleach. Hayward *et al.* [13] reported a 1.8-unit color change after LED light activation, indicating significant tooth whitening. Conversely, Moncado *et al.* [14] found no difference in tooth sensitivity between groups. Bortolatto *et al.* [15] observed similar color and luminosities after treatment in both groups, suggesting that light activation may not significantly impact tooth whitening. Kury *et al.* [16] reported a significant reduction in tooth sensitivity with photocatalytic equipment.

Our study's results are further in line with those of Park *et al.* [17], who found that LED light activation can result in significant tooth whitening. Omidi *et al.* [18] found no significant difference in teeth whitening between groups. Santos *et al.* [19] found no tooth sensitivity reported at 180 days. Zouiten *et al.* [20] reported a whitening effect similar to that observed after 18 months, suggesting that tooth whitening can be maintained over time. The current study's results did not demonstrate a substantial association between light activation and tooth sensitivity during in-office bleaching. This conclusion aligns with the findings of a comprehensive analysis undertaken by Barros *et al.* [21], who discovered limited evidence endorsing the use of light activation in combination with in-office crucial bleaching. Notably, the concentration of hydrogen peroxide utilised did not impact the link between light activation and tooth sensitivity.

In theory, both heat and light sources can accelerate the breakdown of hydrogen peroxide, leading to the production of oxygen and perhydroxy free radicals. It is believed that these reactive species have a role in the bleaching process, possibly enhancing its effectiveness [22-23]. However, the actual mechanisms underpinning this process deserve further exploration. In-office bleaching techniques offer various advantages, including their quickness and relatively minimal danger when conducted by a competent specialist. Nevertheless, they also have several negatives, such as their high cost and uncertain outcomes, which can be impacted by different variables. The ultimate outcome of the bleaching process is determined by an intricate interaction of factors, such as the patient's age, initial shade of tooth colour, concentration of the bleaching chemical, and duration of the therapy. Previous investigations have generated mixed results about the efficiency of light activation in bleaching methods. For instance, Carneiro *et al.* [24] showed that light activation of HP did not give a substantial benefit, as the attained color stability was not maintained beyond 3 months. Furthermore, the increase in temperature associated with light activation may have deleterious consequences on the tooth pulp. Similarly, Cardoso *et al.* [25] discovered that while photoactivation can generate rapid bleaching outcomes, the color reversion was evident in less than a year. These findings underscore the need for future research to completely understand the impact of light activation on bleaching outcomes and to establish effective treatment regimens. The present study was not without its limitations. The small sample size and lack of diversity among the subjects may have restricted the generalizability of the findings. Additionally, the reliance on self-reported data may have introduced biases and inaccuracies. The study's design also precluded the control of extraneous variables, which may have influenced the results. Furthermore, the study's duration may not have been sufficient to capture long-term effects, and the measures used may not have been sensitive enough to detect subtle changes. These limitations underscore the need for future research to build upon and expand these findings.

## Conclusion:

Data shows that no statistically significant variations in shade rankings across all groups. Initial results indicated that all light technologies had similar whitening benefits. However, the six-month evaluations revealed that Group D had more long-lasting and superior whitening outcomes compared to the other groups. Although some groups exhibited greater quality, the findings were not uniformly applicable and were influenced by other factors. The study's findings emphasise the intricacy of the matter and the requirement for careful evaluation of several factors. In conclusion, the results of this study add to the current conversation and provide valuable insights for the creation of practices that are supported by evidence.

## Figures and Tables

**Table 1:** Distribution of Study Subjects by Pre-Operative Shade Rank

**Sr. no.**	**Pre-Proc Rank**			
	**Group A**	**Group B**	**Group C**	**Group D**
1	9	9	15	15
2	5	5	9	15
3	9	5	9	16
4	9	9	9	16
5	12	15	12	9

**Table 2:** Distribution of Study Subjects by 1 month Post-operative Shade Rank

**Sr. no.**	**1month Post-operative Rank**			
	**Group A**	**Group B**	**Group C**	**Group D**
1	9	9	5	5
2	5	2	1	5
3	5	5	5	3
4	5	5	5	5
5	9	5	9	5

**Table 3:** Distribution of Study Subjects by 6 months Post Proc Shade Rank

**Sr. no.**	**6month Post-operative Rank**			
	**Group A**	**Group B**	**Group C**	**Group D**
1	9	9	5	5
2	5	2	1	5
3	9	5	9	3
4	9	9	9	5
5	12	5	12	5

**Table 4:** Intergroup Comparison of Pre Procedure Shade Rank

**Group**	**N**	**Pre Proc Rank**		**Kruskal Wallis Test**	
		**Mean**	SD	**Statistic**	**p-value**
Group A	5	8.8	2.49	7.53	0.057
Group B	5	8.6	4.1		
Group C	5	10.8	2.68		
Group D	5	14.2	2.95		

**Table 5:** Intergroup Comparison of 1 month Post Procedure Shade Rank

**Group**	**N**	**1m Post Proc Rank**		**Kruskal Wallis Test**	
		**Mean**	**SD**	**Statistic**	**p-value**
Group A	5	6.6	2.19	2.49	0.477
Group B	5	5.2	2.49		
Group C	5	5	2.83		
Group D	5	4.6	0.89		
